# Quality of Life among Egyptian Patients with Upper and Lower Limb Amputation: Sex Differences

**DOI:** 10.1155/2014/674323

**Published:** 2014-06-04

**Authors:** Salwa A. Mohammed, Amany M. Shebl

**Affiliations:** ^1^Faculty of Nursing, Fayoum University, Egypt; ^2^Faculty of Nursing, Mansoura University, Egypt

## Abstract

*Background.* Limb amputation is a life-changing event that can cause significant disruptions in many important areas of existence. * Aim of this study.* To evaluate the quality of life (QOL) of patients with limb amputation and identify the factors affecting the quality of life of patients with limb amputation among Egyptian patients. * Research Design.* It was a descriptive exploratory design. * Setting.* The study was conducted in Orthopedics and Surgical Department in Emergency Hospital at Mansoura University Hospitals. * Sample.* A sample of convenience of 100 adult male and female patients who met the inclusion criteria was included. * Tools.* (a) Structured interview questionnaire (SIQ) was used to collect personal data, (b) short form (36) health status questionnaires: this part was utilized to assess the quality of life among Egyptian patients with amputation. * Results.* The result of this study indicates that most participants experienced a change in the quality of life. There is a statistically significant difference between total QOL aspects and each of the following: age, gender, educational level, and type of work. * Conclusion.* Limb amputation tends to cause increased disability for those amputated patients. The age, gender, place of amputation, and marital status are found as statistically significant factors with physical component and psychological component.

## 1. Introduction

Amputation could be described as the removal of a body extremity by surgery or trauma. If amputation is taken as a surgical measure, it is used to control pain or disease process in the affected limb [1]. Amputation is one of the most common acquired disabilities [2]. Amputation can involve either the upper or lower limb and occurs at a variety of levels. Lower limb amputation may be unilateral, involving a single limb, or bilateral, involving both of the lower limbs, and can be performed at a minor or major level.

Østlie et al. [3] noted that upper limb amputations often come as a result of a specific “traumatic injury.” Many researchers stress the importance of the arms and hands. Rybarczyk and Behel [4] write that “arm and hand amputations appear to entail qualitatively different experiences than lower limb amputations for several reasons.” These researchers highlight the vast importance of the arm and the hand for activities such as cooking and holding and for nonverbal communication such as “gesturing and physical contact.” There are many potential causes of amputation; the four primary etiological factors necessitating this procedure are vascular disease and infection, trauma, tumours, and congenital abnormalities [5]. Dysvascularity resulting from cardiovascular disease (CVD) and/or diabetes mellitus is the foremost cause of amputation in most developed countries, followed by trauma [6, 7].

Revicki et al. [8] define QOL as a broad range of human experiences related to one's overall well-being. It implies value based on subjective functioning in comparison with personal expectations and is defined by subjective experiences, states, and perceptions. Quality of life (QOL) is a very important domain in amputated patients. Mucsi [9], in discussing highlighted health related quality of life (HRQL), refers to the subjective perceptions of the effect of a disease or its treatment on one's health and overall QOL. It includes physical, psychological, and social dimensions of health as assessed by the patient. HRQOL can be used to describe the effects of disease and injury on the QOL and the effect of clinical interventions on health and general well-being. Studies have also shown QOL to be highly related to both physical and social aspects of an amputee's life. Therefore, quality of life (QOL) is an important issue for the large number of patients who may need to adapt to severe and chronic disability due to trauma [10, 11].

Amputation can lead people to lose their self-esteem, independence, and/or even employment. In fact, the psychosocial adjustment to limb loss has been compared to coping with the loss of a loved one and it is not uncommon for a person who has experienced an amputation to become depressed [12]. Amputation itself is a change in body structure but has a great influence on many activities, participation in activities, and quality of life [13]. On the other hand, amputation causes a variety of physical and psychosocial challenges including alterations in body image and lifestyle, changes in self-concept, impairments in physical functioning, using prosthesis, and feeling pain [14–16].

Psychosocial impacts on upper limb amputees are outlined in the literature. In a research study conducted by Cheung et al. [17], were suggested that upper limb amputees (compared to lower limb amputees) had higher rates of depression and posttraumatic stress syndrome [4]; also it was argued that poor body image following amputation is correlated with a range of negative outcomes, including increased depression and decreased life satisfaction, quality of life, activity levels, and overall psychological adjustment. The functional ability of the individual is often adversely affected, and it has a negative effect on productivity and social engagement [18]. These affect the ability of the person to return to and maintain work, maintain social relationships, participate in leisure activities and be active members of the community [19].

## 2. Aim of the Study

The aim of this study was to evaluate the quality of life (QOL) of patients with amputation and identify the factors affecting the quality of life among Egyptian patients with amputation.

## 3. Research Questions

The following research questions were formulated to achieve the aim of the study.

- What is quality of life among patients with limb amputation?
- What are the factors affecting the quality of life among amputation patients?

## 4. Subjects and Methods

### 4.1. Design

A descriptive and correlational research design was utilized.

### 4.2. Sample

Sample of convenience of 100 male and female patients aged between 18 and 60 years was admitted to the hospital and diagnosed with limb amputation. These patients were adults of both sexes who had undergone primary amputation of the upper and lower extremity at the level of hand, upper arm, foot, lower leg, or upper leg and were aged between 18 and 60 years. They gave their consent to participate in the study. Participants were to be excluded from the current study if they had shown any current musculoskeletal injuries.

### 4.3. Setting

The study was conducted in Orthopedics and Surgical Department and Emergency Hospital at Mansoura University Hospitals, Egypt.

### 4.4. Tools

They were designed by researchers based on literature review; they have included two parts.

#### 4.4.1. Part 1: The Structured Interview Questionnaire (SIQ)

To record patient's sociodemographic data, it was comprised of data related to patient's age, sex, level of education, marital status, and occupation. Also, the part of “patient's medical data” was formulated to assess the patient's health history, for example, site of amputation, causes, and comorbidity associated with treatment.

#### 4.4.2. Part 2: Short Form (SF-36) Health Status Questionnaires

To assess quality of life. The SF-36 was developed by Ware and translated into Arabic by researchers [20]. It consists of 36 questions (items) measuring physical and mental health status in relation to eight health concepts: physical functioning, role limitations due to physical problems, bodily pain, general health perceptions, vitality, social functioning, role limitations due to emotional problems, and mental health. The values of each sub score are computed on a scale from 0 to 100. The raw scale scores from global quality of life were linearly converted to a range of 0 (worst possible health status or quality of life) to 100 (best possible health status or quality of life). The score of the subgroups and all eight scales, as well as the final global score, of the SF-36 range between 0 and 100, indicating that the lower the score the more the disability and the higher the score the less the disability.

### 4.5. Ethical Consideration

The aim of the study was explained to patients and a written/oral consent will be obtained before asking them to participate in the study after ensuring the confidentiality of the collected information, and the patient was free to withdraw at any time of the study.

### 4.6. Validity and Reliability

The developed questionnaires tools were reviewed by five panels of experts in medical surgical nursing in order to ensure content comprehensiveness, clarity, relevancy, and applicability. The test-retest reliability coefficient for the total SF-36 was 86.5. The questionnaires were translated from English into Arabic to help the patient understand them.

### 4.7. Pilot Study

A pilot study was carried out on 10 patients to test feasibility, objectivity, and applicability of the study tools. Based on the results of the pilot study, the needed refinements and modifications were made.

## 5. Procedure

An official permission was obtained from the hospital administrative authority after explaining the aim of the study. The researchers met the selected patient preoperatively. The purpose and nature of the study were explained and the patient consent was obtained. Baseline data, which were established using the structured interview questionnaire and SF-36 sheet for measuring the quality of life for patients, were read and were also explained. The patients' answers were recorded by the researchers. Each participant was interviewed individually and the data collection time for each patient lasted for almost 15 to 30 minutes. The process of data collection for this study took place during the period from January to May 2013.

## 6. Data Analysis

The data were analyzed using SPSS (version 12). The QOL scores, the clinical results, and the demographic characteristics of the participants were summarized using the descriptive statistics of frequency, mean, standard deviation, and percentages as appropriate. Mann-Whitney *U* test was used to compare both overall and domain QOL scores of male and female participants. Pearson's correlation test was used. Statistical significance was considered at *P* value < 0.05.

## 7. Results

**Figure 1:**
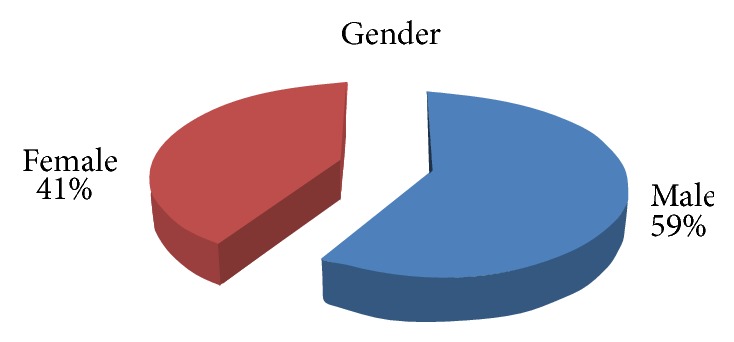
Gender of subjects in the sample. Percentage of distribution of studied sample in relation to gender (*n* = 100).

*Figure 1: Distribution of Gender of Subjects in Study Sample*. This figure indicates that more than half of the sample (59%) was male and nearly two-fifths of the sample was female (41%).

*Table 1: Distribution of Sociodemographic Characteristics of the Study Sample*. This table presents that more than half of the females (53.65%) and about two-fifths of the males (44.06%) in age group ranged from fifty to less than sixty years with means 47.61 and 48.20, respectively. Regarding their marital status, most of the males (76.82%) and most of the females (60.97%) were married. For the level of education, nearly two-fifths of the males (42.37%) and over one-third of the females (38.1%) are illiterate. In relation to type of work, most of the males and most of the females (67.79 and 73.17%) are having jobs that require physical efforts. Regarding residence, most of the males and females (50.84% and 53.65%, resp.) are from rural areas. Most of the males and females (69.49 and 95.12%) live with their families. Finally, regarding income, more than half of the males and females (61.01% and 65.85%, resp.) have unsatisfactory income.

*Table 2: Distribution of Clinical Characteristics of the Sample*. This table shows that amputations in more than half of the males (50.84%) and more than half of the females (51.21%) were caused by disease of diabetes while nearly in one-third of males and females (30.50 and 39.02) they were caused by vascular disease. Regarding the place of amputation, most of the males and females (52.54% and 53.65%, resp.) have amputation in the lower limb. Finally, as for comorbidity, more than half of the sample has no comorbid disease.

*Table 3: Measurement of Central Tendency and Distribution of Quality of Life among Sample.* This table shows that male participation in physical component summary (mean=65.53 and 53.32, *P* = 0.042), physical functioning (46.82 and 35.62, *P* = 026) and emotional role (53.10 and 64.21, *P* = 0.34) scored significantly higher than female participation respectively.

*Table 4: Described Correlation of Some Research Variables and Dimensions of Quality of Life among Patients*. This table shows that female subjects had significantly higher mean scores than male in relation to physical component and mental component (*r*=0.028, 0.042, *P* ≤ 0.05, resp.). Also there are positive correlation between marital status (married) and mental components(*r*=0.05, *P* ≤ 0.05, resp.) this may be attributed to husband/family provided social support. In relation to age, there are statistically significant negative relations with mental health. Regarding site of amputation, also there are significant statistics in relation to upper limb and physical component (0.043, *P* < 0.05, resp.) in addition to lower limb and mental component (0.034, *P* < 0.05, respectively). No statistically significant relation is found among them regarding educational level, residence and causes of amputation.

## 8. Discussion

Amputation has become one of the common problems in the present society. A number of people have one or both limbs amputated and the situation moves to an increase worldwide. Traumatic amputation is a catastrophic work injury and often a major cause of disability [1]. Individuals with an amputation have to adapt to several losses and changes to their lifestyle, social interactions, and identity [21]. Therefore, the current study aims to assess QOL and to determine the factors affecting QOL of patients with amputation.

Our study showed that males constituted 59% of the participants in this study and females 41%. The majority of amputations were in the lower limb. This supports findings from previous studies that lower limb amputations are more common among males than females [22–24]. Also The Global Lower Extremity Study [25] stated that the incidence of LLA is similar in females and males in some regions and higher in females compared to males in other regions although the overall incidence is higher in males than females.

In this study, the mean age of amputation due to diabetes, trauma, and vascular disease was 47.84 years, respectively. The results are comparable to other studies which showed that the majority of patients are males with range of 14–65 years (mean age: 33.29 years) which means that it most commonly involves the reproductive age group [26]. Desmond [27] showed that the majority of the patients ranged from 11 to 52 years old. In addition, results of Marzen-Groller and Bartman [28] indicated that the majority (75%) of amputations occur in people who are aged more than 65 years.

Diabetes mellitus was found to be the leading cause of amputation in this study. This result is similar to those previous studies performed by Johannesson et al. [29], which reported that individuals with diabetes have a significantly elevated rate of amputation when compared to individuals without diabetes. Increasing amputation rates among individuals with diabetes have been attributed to the fact that the persons with diabetes have poor level of knowledge about diabetes and diabetic foot care. This had contributed to an increase in the average age at which amputation occurs. In contrast, a study by Moxey et al. [30] found that 39% of patients who underwent major amputations within the time span of five years in England had a primary diagnosis of diabetes, and 43% had a diagnosis of CVD, with just 13.9% of procedures being secondary to injury or trauma. These results support the findings that 54% of all existing cases of limb loss in the USA are secondary to vascular disease, two-thirds of which also involve a comorbid diagnosis of diabetes (Ziegler-Graham et al. [7]). In addition to the current literature, amputations also result from military combat or other types of violence [31]. This study also reveals that the majority of all participants' amputation occurred in lower limb. This result is similar to those of previous studies performed by Ziegler-Graham et al. [7], and National Amputee Statistical Database [6] indicated that lower limb amputation is significantly more common than amputation of the upper limb; also it revealed that amputations of lower limbs occur in significantly greater numbers than do amputations of upper limbs. This result is further supported by Tseng et al. [32].

People with lower limb amputation had worse QOL as compared to the general population. Results of the current study supported that amputation continued to be associated with poorer quality of life over some dimension for male and female. These were demonstrated by physical functioning activities, physical role, and bodily pain. This finding is consistent with previous research; Demet et al.'s [33] study revealed that upper limb amputees' high reported QOL (compared to lower limb amputees) is primarily related to their responses pertaining to “physical disability, pain, and energy level.” Dunn [34] found that younger amputees are significantly more at risk of developing depression than older amputees on account of activity restriction.

This result is similar to those of previous studies performed by Zidarov et al. [35] which report that all participants had poor scores of physical functions (ability to go outside and overall fitness) at baseline and remained poor at three-month follow-up. The study results of Sinha et al. [36] among (*n* = 605) limb amputated patients are on the same line. This finding is considered to be the most important factor influencing the physical health component of QOL, whereas the employment status and comorbidities impacted mainly the mental health component of QOL in amputees.

In the present study, no statistically significant relations are detected among (SF-36) dimension scores, causes of amputation, and residence. This is not surprising and can be referred to from the small size of the sample. Results of this study also show that there are statistically significant relations between age and mental health component. These results are comparable to those of a study by Dunn [34] which finds that when amputation occurred in young person, higher levels of depression are reported. Another study on recent and long-term amputees, who belong to either young or old age group, found that, in older group, the longer the time since amputation is, the fewer the psychological symptoms and less depression are exhibited. Younger amputees had increased psychological symptoms and increased rate of depression. Younger amputees appear to be anxious, sensitized, vigilant persons who had difficulty in integrating their present life. Frank et al. [37] and Shabaan et al. [38] also report that there is a statistically significant association between psychological status and patient age. In the recent literature, another study done by Goals [39] over 113 patients during the period following accident, illness, or injury found that age, gender, and cause of amputation are significantly associated with the psychological status.

Moreover, the result of the present study revealed that there is a statistically significant difference between marital status and psychological aspect. This may be attributed to social support from family. Regarding patient sex, this study shows that there is a statistically significant difference between sex and total patient's QOL. This is consistent with a study by Williams and associates [40] which proves that being a female is a significant predictor of greater symptoms of depression at six months after amputation. In addition, some longitudinal studies have failed to observe significant changes in psychosocial outcomes over time among persons with amputations. This distribution is in agreement with another study carried out by Demet and colleagues [34] who reveal that younger individuals with upper or lower limb amputations have a higher QOL in several domains, including emotional reactions and social isolation. In the same line a study carried out by Deans and associates [41], which examined QOL in 75 individuals with above- or below-knee amputations, indicated that QOL in the physical domain is affected the most in this patient group.

## 9. Conclusions and Recommendation

Based on the findings of the present study, it can be concluded that the quality of life automatically drops after losing any important part of one's body. The most affected aspects are the physical and mental ones and this is very frequent in amputation. The age, gender, place of amputation, and marital status are found as statistically significant factors with physical and psychological components, while there was no statistically significant difference among QOL aspects, educational level, type of work, residence, and living accommodation. It is recommended that the participants receive a structured rehabilitation program which is appropriate to the specific needs of people with limb amputation in order to be able to find out its impact on their functional status and QOL. Also replication of the study on a larger sample from different geographical areas should be done to achieve more general results.

## 10. Limitations

Small size of sample and QOL of individuals with limb amputation were not compared with those of age- and sex-matched controls, so the study findings cannot be generalized. Another limitation of this study was that there is a need for prospective longitudinal studies to systematically follow the change in the QOL of individuals with limb amputation over time and assess its determinants.

## Figures and Tables

**Table 1:** Sociodemographic characteristics of the studied amputation patients.

Characteristics	Male (*n* = 59)		Female (*n* = 41)		Total (*n* = 100)	
	No	%	No	%	No	%
Age in years						
<40	13	22.03	2	4.87	15	15
40–49	19	32.20	17	41.46	36	36
50–59	26	44.06	22	53.65	48	48
59+	1	1.69	0	0	1	1
Mean ± SD	48.20 ± 12.92		47.61 ± 9.86		47.82 ± 11.53	
Marital status						
Single	3	5.08	1	2.43	4	4
Married	45	76.82	25	60.97	70	70
Divorced	6	10.16	2	4.87	8	8
Widowed	5	8.47	13	31.70	18	18
Educational level						
Illiterate	13	22.03	8	38.1	21	21
Primary	16	27.11	15	23.8	31	21
Secondary	25	42.37	15	23.8	40	40
High	5	8.47	3	14.3	8	8
Type of work						
Mental	19	32.20	11	26.82	31	32
Physical	40	67.79	30	73.17	69	68
Residence						
Urban	29	49.15	19	46.34	48	48
Rural	30	50.84	22	53.65	52	52
Living accommodation						
With family	41	69.49	39	95.12	80	80
With relatives	18	30.50	2	4.87	20	20
Alone	0	0	0	0	0	0
Monthly income						
Satisfaction	23	33.98	14	34.14	37	37
Unsatisfaction	36	61.01	27	65.85	63	63

**Table 2:** Clinical characteristics of the sample.

Characteristics	Male (*n* = 59)		Female (*n* = 41)		Total (*n* = 50)	
	No	%	No	%	No	%
Causes of amputation						
Vascular	18	30.50	16	39.02	34	34
Diabetes	30	50.84	21	51.21	51	51
Accident	11	18.64	3	7.31	14	14
Others	0	0	1	2.43	1	1
Place of amputation						
Upper limb	28	47.45	19	46.34	47	47
Lower limb	31	52.54	22	53.65	53	53
Comorbidity						
Yes	29	49.15	16	39.02	45	45
No	30	50.84	25	60.97	55	55

**Table 3:** Measurement of central tendency and distribution of quality of life among sample.

Variable	Male Mean (SD) (*n* = 59)	Female Mean (SD) (*n* = 41)	*P* value
Role physical (RP)	34.56 (30.60)	30.51 (29.78)	0.630
Physical functioning (PF)	46.82 (23.95)	35.62 (25.56)	0.026∗
Bodily pain (BP)	41.74 (23.22)	43.15 (24.13)	0.872
Energy/fatigue/vitality	68.05 (16.09)	62.12 (18.03)	0.781
Mental health (MH)	50.42 (12.67)	46.31 (20.13)	0.532
Role emotional	53.10 (35.65)	64.21 (31.15)	0.034∗
General health	57.13 (14.17)	52.12 (16.25)	0.802
Physical component summary (PCS)	65.53 (13.51)	53.32 (14.23)	0.042∗
Mental component summary (MCS)	63.22 (14.72)	60.31 (15.31)	0.712

^*^No significance at *P* > 0.05.

**Table 4:** Correlation of some research variables and dimensions of quality of life among patients.

Research variable	Quality of life dimensions (SF-36)	
	Physical component	Mental component
Age		
<40 years	0.283	−0.021∗
≥40 years	−0.580	0.561
Gender		
(i) Male	0.065	0.216
(ii) Female	0.028∗	0.042∗
Marital status		
(i) Married	0.293	0.05∗
(ii) Not married	0.314	0.282
Residence		
(i) Rural	0.20	0.07
(ii) Urban	0.462	0.49
Educational level		
(i) Illiterate	0.315	0.154
(ii) Literate	0.213	0.104∗
Causes of amputation		
(i) Vascular	0.54	0.48
(ii) Nonvascular	0.49	0.62
Site of amputation		
(i) Upper limb	0.044∗	0.073
(ii) Lower limb	0.161	0.034∗

^*^Significance at *P* < 0.05.
